# Anomalous behavior above the Curie temperature in (Nd_1−*x*_Gd_*x*_)_0.55_Sr_0.45_MnO_3_ (*x* = 0, 0.1, 0.3 and 0.5)

**DOI:** 10.1039/c9ra03303c

**Published:** 2019-09-02

**Authors:** R. Kamel, A. Tozri, E. Dhahri, E. K. Hlil

**Affiliations:** Laboratoire de Physique Appliquée, Faculté des Sciences de Sfax, Université de Sfax B. P. 1171 Sfax 3000 Tunisia ra7ma_7@hotmail.fr; Physics Department, College of Science, Jouf University P. O. Box: 2014 Sakaka Saudi Arabia; Institut Néel, CNRS – Université J. Fourier B. P. 166 38042 Grenoble France

## Abstract

Magnetic properties were studied just above the ferromagnetic–paramagnetic (FM–PM) phase transition of (Nd_1−*x*_Gd_*x*_)_0.55_Sr_0.45_MnO_3_ with *x* = 0, 0.1, 0.3 and 0.5. The low-field inverse susceptibility (*χ*^−1^) of Nd_0.55_Sr_0.45_MnO_3_ exhibits a Curie–Weiss-PM behavior. For *x* ≥ 0.1, we observe a deviation in *χ*^−1^(*T*) behavior from the Curie–Weiss law. The anomalous behavior of the *χ*^−1^(*T*) was qualified as Griffiths phase (GP)-like. The study of the evolution of the GP through a susceptibility exponent, the GP temperature and the temperature range of the GP reveals that the origin of the GP is primary due to the accommodated strain. Likewise, the magnetic data reveal distinct features visible only for *x* = 0.5 at a low magnetic field that can be qualitatively understood as the result of ferromagnetic polarons, entailed by the strong effect of chemical/structural disorder, whose concentration increases upon cooling towards the Curie temperature. We explained the magnetic properties at a high temperature for the heavily Gd-doped sample (*x* = 0.5) within the phase-separation scenario as an assembly of ferromagnetic nanodomains, antiferromagnetically coupled by correlated Jahn–Teller polarons.

## Abstract 2

Magnetic properties were studied just above the ferromagnetic–paramagnetic (FM–PM) phase transition of (Nd_1−*x*_Gd_*x*_)_0.55_Sr_0.45_MnO_3_ with *x* = 0, 0.1, 0.3 and 0.5.

## Graphical abstract

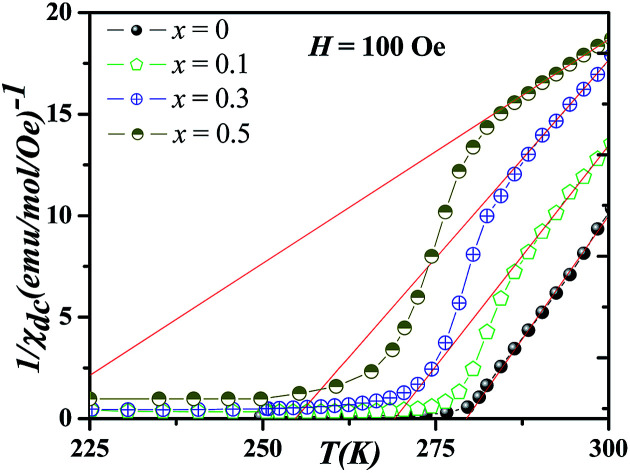

## I. Introduction

The phenomenon of colossal magnetoresistance (CMR) in ABO_3_-type perovskite manganites with the generic formula Re_1−*x*_Ae_*x*_MnO_3_ (Re is a trivalent rare-earth ion and Ae is a divalent alkaline-earth metal ion) has attracted considerable attention in recent years.^1^ These systems are currently viewed as important examples of strongly correlated electronic systems offering a unique combination of coupling between charge, spin, orbital and lattice degrees of freedom, which results in a great variety and complexity of physical phenomena and an observation of phase transition.

One of the main results of the research into manganites is the discovery of a strong tendency toward phase separation, *i.e*., the formation of inhomogeneous states of nano- to micro-meter length scales in the form of coexisting competing ferromagnetic (FM) and antiferromagnetic (AFM)/paramagnetic phases (PM) which support charge and orbital order. This robust result, appears both in experimental and in theoretical models.^2^ Recently, the phase separation has been confirmed to be intrinsic and crucial to the comprehension of the CMR effect and the related properties in manganite.^2^ The origin of phase separation has been attributed to various reasons such as quenched disorder by chemical doping, the random field, and the localized and broad electronic wave functions (L–B model).^2,3^ It has been suggested that different crystal structures of the coexistence phases (*i.e*. FMM and AFM–COI) generate long-range strain interactions leading to an intrinsic variation in elastic energy landscape, which in turn leads to phase separation.^4,5^

Among the various forms of phase separation, the concept of preformation of FM ordered clusters at some well defined temperature T* much above the FM long-range ordering temperature (*T*_C_) (*i.e*., still in the PM phase) is a widely accepted phenomenon.^6^ This regime between T* and *T*_C_, was predicted by Griffiths^7^ in diluted Ising ferromagnets and has since generated vested interests.^8^ In its simplest form, the original problem considered the percolative nature of an Ising system to have the nearest neighbor (exchange) bonds characterized by a strength *J* occurring with a probability *p*; otherwise, the bond strength is zero. For *p* < *p*_c_ (percolation threshold), an infinite percolating backbone cannot be formed (or, equivalently, the correlation length does not diverge) and thus no cooperative FM transition occurs. Above *p*_c_, the FM phase exists in a weakened form by the shortage of a percolation path; hence, thermal fluctuations will destroy the FM phase at a temperature *T*_C_, which is lower than the critical temperature *T*_G_ (=T* > *T*_C_) of the pure FM phase (Griffiths temperature). The effect of disorder above *T*_C_ is to destabilize the pure system into small FM clusters. These small clusters give rise to characteristic features that allow the identification of the so-called Griffiths phase (GP), namely, the deviation of the reciprocal susceptibility (*χ*^−1^) from the Curie–Weiss (CW) predictions as the system approaches *T*_C_ (on cooling, from *T* > *T*_G_), taking the form of an enhanced low-field susceptibility.^7^ Bray generalized this argument for any bond distribution (instead of bonds having strength of only *J* and 0) which reduces the long-range ordering transition temperature.^9^ The presence of the Griffiths singularity has been widely observed in various systems, including magnetic semiconductors,^10^ intermetallic,^11^ rare-earth compounds,^12^ cobaltite,^13^ and hole-doped perovskite manganite.^14–21^ The appearance of the GP is usually associated with competing magnetic interactions leading to FM clustering, the origin of which may differ from one material to another.

In CMR systems, quenched disorder is argued to lead to a distribution of exchange energies that cause Griffiths-like behavior.^14^ Indeed, the substitution at the A-site cations inevitably introduces quenched disorder as well since the systems are solid solution compounds. Random distribution of different A-site cations leads to a structural and electronic disorder. The quenched disorder associated with the solid solution of the A-site cations is quantified using the ionic radius variance 
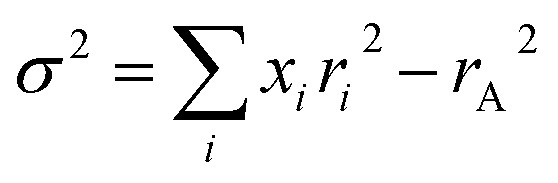
, where *x*_*i*_ and *r*_*i*_ are the fractional occupancies and the effective ionic radii of cations of Re and Ae, respectively.^22^ In several reports, it was claimed that CMR effect is associated with the GP driven by intrinsic randomness, the combined effect of doping, the tendency for charge segregation and the self-trapping effect associated with polaron formation.^14–17^ Nevertheless, Tong *et al.*^20^ and Jiang *et al.*^23^ reported that the GP account for CMR is not a prerequisite in manganites. Until now, there is a debate on whether the GP is always a precursor to CMR in manganites or not.

The aim of this brief report is to investigate the effect of the isovalent substitutions of the larger Nd^3+^ (^IX^*r*_Nd_^3+^ = 1.163 Å) by the smaller Gd^3+^ in Nd_0.55_Sr_0.45_MnO_3_ on the magnetic behavior above *T*_C_. The solid solution (Nd_1−*x*_Gd_*x*_)_0.55_Sr_0.45_MnO_3_ (*x* = 0, 0.1, 0.3 and 0.5), with constant Mn^3+^/Mn^4+^ ratio, is based on a strongly-magnetic heavy rare-earth [Gd^3+^ (*L* = 0 and *S* = 7/2)] of an intrinsically large magnetic moment and small ionic radius (Gd: *μ*_eff_ = 7.94 *μ*_B_, ^IX^*r*_Gd_^3+^ = 1.107 Å). Here we study the observed downturn in *χ*^−1^ in (Nd_1−*x*_Gd_*x*_)_0.55_Sr_0.45_MnO_3_, its origin, and its progressive development on addition of quenched disorder at the A-site by substitution of magnetic Gd^3+^ (4f^7^). Our experiments demonstrate that the origin of the Griffiths phase is probably associated with the accommodation strain in addition to the quenched disorders. However, the data reveal distinct features visible only for *x* = 0.5 at low magnetic field that can be qualitatively understood as the result of ferromagnetic polarons which claim that GP seems to be doubtful or at least coexists with the ferromagnetic polarons.

## II. Experimental details

Samples of (Nd_1−*x*_Gd_*x*_)_0.55_Sr_0.45_MnO_3_ (NGSMO) series with *x* = 0, 0.1, 0.3 and 0.5 are studied here. They are prepared by standard solid-state ceramic route and characterized by various techniques such as Rietveld profile refinement of X-ray diffraction data and microprobe analysis.^24^ For *x* = 0.5, the best fitting results were obtained upon using the model of two distinct orthorhombic *Pnma* phases with close lattice parameters. DC magnetization was measured with a home-made BS2 magnetometer developed at Néel Institute (Grenoble-France). This magnetometer use extraction technique and can produce a field of 10 T. It is found that the undoped sample (*x* = 0) undergoes a sharp PM–FM transition at *T*_C1_ ∼ 276 K. While for *x* ≥ 0.1 samples, two FM transitions at *T*_C1_ which decrease with *x* and *T*_C2_ ∼ 70 K are observed, respectively.^24^ The *T*_C_ for all the samples are deduced from the inflection point of low-field dc-magnetic data.^24^

## III. Results and discussion

**Fig. 1:**
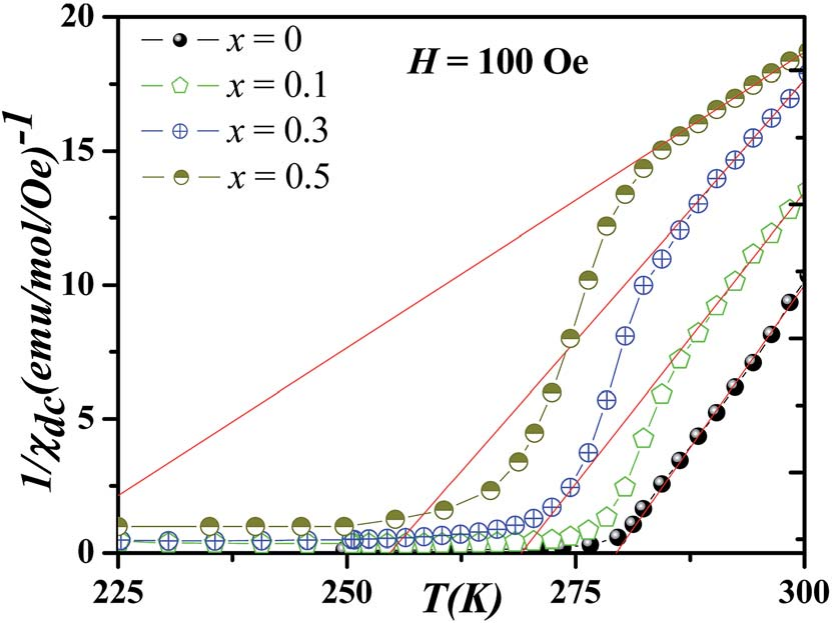
Thermal dependence of inverse susceptibility (*χ*^−1^ = *H*/*M*) of the compounds at *H* = 100 Oe. Solid lines are Curie–Weiss fits to the high temperature behavior.

Fig. 1 illustrates the temperature (*T*) dependencies of the low-field reciprocal susceptibility (*χ*^−1^ = *H*/*M*) at 100 Oe for NGSMO. It is well known that in the PM region, the relation between *χ* and *T* should follow the CW law, *i.e*. *χ* = *C*/(*T* − *θ*_p_), where *C* is the Curie constant and *θ*_p_ is the Weiss temperature. The line in Fig. 1 is the best fitting curve deduced from the CW equation. The effective magnetic moment *μ*_eff_ obtained from the fitted *C*, and the fitted *θ*_p_ are shown in Table 1.

**Table 1:** Magnetic data of the (Nd_1−*x*_Gd_*x*_)_0.55_Sr_0.45_MnO_3_ solid solution

*x* (Gd)	*T*_C1_^24^	*T*_C2_ (ref. 24)	*θ*_*p*_/K^a^	*μ* ^exp^_eff_ (tot)/*μ*_B_	*μ*_Mn_/*μ*_B_^b^	*μ*_Nd_/*μ*_B_^c^	*μ*_Gd_/*μ*_B_^d^
0	276 ± 0.2	—	279	3.98	4.621	2.774	0
0.1	271 ± 0.2	70.38 ± 0.2	269	4.23	4.553	2.632	1.862
0.3	261 ± 0.2	70 ± 0.2	255	4.49	4.535	2.321	3.225
0.5	260.8 ± 0.2	75 ± 0.2	206	5.92	4.915	1.962	4.163

a Extrapolation at *χ*^−1^ = 0 of a Curie–Weiss behaviour.

b Magnetic contribution of the Mn sublattice, deduced from eqn (1).

c Assuming *μ*_Nd_ = 3.74/*μ*_B_^−1^.

d Assuming *μ*_Gd_ = 7.94/*μ*_B_^−1^.

For the pristine sample, *χ* perfectly follows the CW law at temperatures just above *T*_C1_, *i.e. χ*^−1^ is linearly dependent on *T*, which is characteristic of homogeneous paramagnets. This is confirmed by the value of *θ*_p_ (∼279 K) which is very close to *T*_C1_ (∼276 K). However, *χ*^−1^ for *x* ≥ 0.1 exhibits a CW-PM behavior only at high *T*, *i.e*. *χ*^−1^ show a linear dependence on temperature for *T* > 280 K. Upon cooling, it is interesting to find that *χ*^−1^(*T*) show a deviation from the CW law well above *T*_C1_, which strongly suggests that a short-range FM state is formed before the long-range FM transitions in those compounds. Though the decrease in *θ*_p_ [Table 1] indicates that the FM interactions are weakened by Gd doping, all compositions show positive value of *θ*_p_ indicating that FM interactions are dominant in these compounds. In particular, for *x* = 0.5, the *θ*_p_ is far below the long-rang ordering temperature *T*_C1_. In agreement with reports pertaining to isovalent substitution in manganites, such as La_1−*x*_Gd_*x*_MnO_3_,^25^*θ*_p_ is determined by the sum of AFM and FM interaction. These coexisting interactions decrease the value of *θ*_p_ much below the ordering temperature.

In general, the PM regime is observed at a high temperature with fairly parallels thermal dependences for all samples, indicating an almost constant effective moment *μ*_eff_. If we suppose that the PM regime refers simply to the superposition of the Mn and the Re sublattices, then their respective contributions add up in the following way: *C*_tot_ = *C*(Nd) + *C*(Gd) + *C*(Mn) where *C*(*i*) is the Curie constant of the *i*-sublattice. The magnetic moment of the transition-metal (Mn) contribution is then evaluated upon the following relation:

1 *μ*_Mn_ = [*μ*_eff_^2^ − (*μ*_Gd_^2^ + *μ*_Nd_^2^)]^1/2^

where *μ*_Gd_ is the contribution of gadolinium (*μ*(Gd^3+^) = 7.94 *μ*_B_) normalized to its concentration (*μ*_Gd_^2^ = (7.94)^2^(0.55 × *x*) *μ*_B_^2^) and *μ*_Nd_ is the contribution of neodymium (*μ*(Nd^3+^) = 3.74 *μ*_B_) normalized to its concentration (*μ*_Nd_^2^ = (3.74)^2^(0.55 × (1 − *x*)) *μ*_B_^2^). Table 1 gives the contribution of Gd and Nd for each composition NGSMO, together with the Mn contributions calculated from eqn (1). The increase of the overall magnetic moment is actually due to the increase of the total number of gadolinium atom during the Nd → Gd substitution, while the magnetic moment of the Mn sublattice stays relatively constant (4.4–4.6 *μ*_B_). The resulting magnetic moment [Table 1] which corresponds to contribution of the transition-metal Mn is in general close to the theoretically expected PM moment of the Mn spins (*i.e.* calculated for free Mn^3+^ (*S* = 2) and Mn^4+^ (*S* = 3/2) considering a spin-only contribution) deduced from: 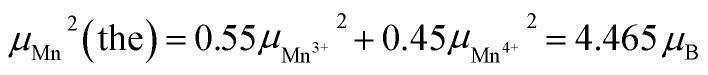, where *μ*_Mn^3+^_ = 4.98 *μ*_B_ and *μ*_Mn^4+^_ = 3.87 *μ*_B_.

For *x* ≥ 0.1 samples, *χ*^−1^ is CW-like at high *T*, but upon cooling, *χ*^−1^(*T*) shows departures from the high-*T* CW behavior at *T* > *T*_C1_. Note that the result we come up with is that the data deviate downward from the CW prediction. This downturn in *χ*^−1^ above *T*_C1_ is an important observation which distinguishes the GP from smeared phase transition. In fact, the latter case gives rises to an upward curvature in *χ*^−1^(*T*) above *T*_C_, deviating from CW behavior.^26^ While the GP is characterized as divergence in susceptibility, which implies that *χ*^−1^ would exhibit a sharp downturn with decreasing temperature.^27^ Hence, for *x* ≥ 0.1 samples, all *χ*^−1^(*T*) curves reveal a GP-like downturn below a certain temperature. The onset of this downturn is denoted as *T*_G_ (*i.e*., the temperature where *χ*^−1^(*T*) deviates from the CW behavior) below which the FM clusters emerge in the PM matrix, as is described in a GP system.^28^ By contrast, for Nd_0.55_Sr_0.45_MnO_3_ (*x* = 0), within the limits of experimental error, there is no anomaly occurring as deviations of the curves *χ*^−1^(*T*) at temperature *T* > *T*_C1_ from the CW law. Such behavior points out that the Griffiths singularity or the smeared phase transition is completely absent in the corresponding data.

**Fig. 2:**
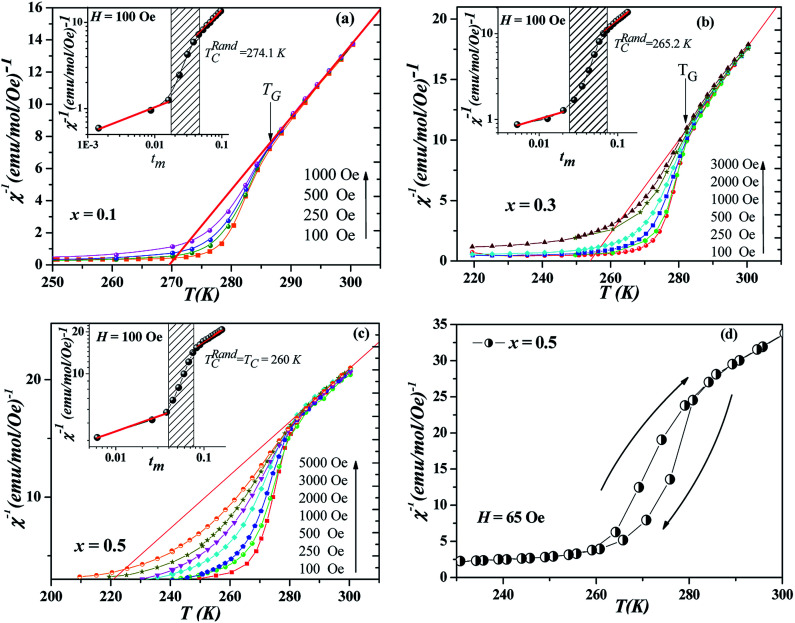
Temperature variation in *χ*^−1^ measured at different magnetic fields of (a) *x* = 0.1, (b) *x* = 0.3 and (c) *x* = 0.5. Arrows indicate the Griffiths temperature *T*_G_ (see text for its detailed definition). The insets replot these data on a double-logarithmic scale, testing the power law eqn (2) with reduced temperature *t*_m_ = (*T* − *T*^Rand^_C_)/*T*^Rand^_C_ and yielding estimates for exponent *λ*_GP_ and *λ*_PM_. Panel (d) illustrates the hysteresis in *χ*^−1^ at 65 Oe for *x* = 0.5. Solid lines are Curie–Weiss fit indicating that the downturn vanishes at high *H* transforming to normal CW behaviour.

The softening of the downturn in *χ*^−1^ with the progressive increase in magnetic field (*H*) is a typical signature of GP which has also been observed in a variety of other systems.^16–21,28^ For further confirmation that the observed anomaly in *x* > 0.1 is actually due to the Griffiths singularity, we have measured *χ* for different *H* [Fig. 2(a)–(c)]. It is clear from these figures that at a lowest measuring field of 100 Oe, the downturn is reasonably sharp, while with increasing field, the sharpness of downturn is reduced. Indeed, the downturn vanishes at a high field and the *χ*^−1^(*T*) fully obeys the conventional CW law in the PM state. Based on the original paper of Griffiths^7^ and subsequent work,^9,16–21,28^ this behavior is another characteristic of GP, and this is due to the polarization of spins outside the clusters or in terms of the masking of the FM signal by the rising PM background, as already proposed by Deisenhofer *et al.*^16^ in manganite systems. We emphasize that the magnetic field required to remove this anomaly increase with Gd doping; from 1 kOe for *x* = 0.1 up to 5 kOe for *x* = 0.5, indicating the increase of the strength of the GP with the increase of the Gd content.

**Fig. 3:**
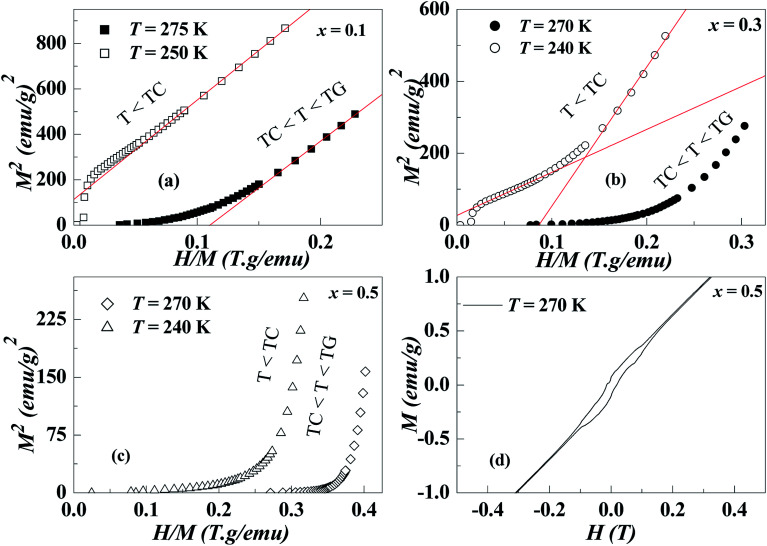
(a), (b) and (c) Arrott plot (*M*^2^*vs. H*/*M*) of isotherms collected at different temperatures both below and above *T*_C_ are plotted for *x* = 0.1, 0.3 and 0.5, respectively. Lines are due to straight-line fitting of plot in high field. Arrows indicate the spontaneous magnetization. (d) Hysteresis loops for *x* = 0.5 measured at *T*_C_ < *T* = 270 K < *T*_G_.

Among the important characteristics of GP is that the system as a whole would not develop static long-range order, as such it would not exhibit any spontaneous magnetization (*M*_S_ is zero) which is the opposite in the case of smeared phase region.^8^ Though in the GP regime *T*_C_ < *T* < *T*_G_ there exists a finite-size cluster with FM correlated spins. To verify this, *M*(*H*) has been measured both below and above *T*_C1_. Fig. 3(a)–(c) show the Arrott plots [*M*^2^*vs. H*/*M*] (only a few of such isotherms are shown for clarity).^29^ Intercept on positive *M*^2^ axis of the high-field extrapolation of this plot gives *M*_S_. Fig. 3(a) and (b) demonstrate that nonzero *M*_S_ exists only below *T*_C1_ which unambiguously show that in *x* = 0.1 and 0.3, the phase above *T*_C1_ is GP and not pure PM. However, for *x* = 0.5 [Fig. 3(c)], none of the curves show a FM nature indicating the absence of spontaneous magnetization. A self-consistent approach of analyzing modified Arrott plots may not be applicable here (*x* = 0.5) since it produces unphysical scaling constants.^21^ In order to further understand the magnetization of *x* = 0.5 samples, measurement of hysteresis loops of magnetization shown in Fig. 3(d) was performed at 270 K. From this figure, the occurrence of ferromagnetism is confirmed in the region *T*_C1_ < *T* < *T*_G_ with lower coercivity (∼5 Oe).

According to the model of the GP, the system exhibits neither a pure PM behavior nor a long-range FM order in the GP regime.^7–9^ Consequently, the system response is dominated by the largest magnetic cluster/correlated volume, which will give rise to a characteristic *T*-dependence for the low-field *χ*^−1^ by the following power law:^27^

2 *χ*^−1^(*T*) ∝ (*T* − *T*^Rand^_C_)^1−*λ*^

where 0 ≤ λ < 1 and *T*^Rand^_C_ is the critical temperature of random FM where susceptibility tends to diverge.

It is clear that the power law behavior in eqn (2) is a modified CW law where the exponent *λ* quantifies a deviation from CW behavior due to the formation of magnetic clusters in PM state. As *T*_C_ is approached from above, more number of clusters achieve FM ordering and the bulk susceptibility of system tends to diverge at *T*^Rand^_C_ which is defined to lie above the actual ordering temperature but below the highest ordering temperature allowed by the exchange bond distribution, *T*_G_.^7,9,29^ In order to further confirm the Griffiths singularity in NGSMO, we have fitted *χ*^−1^(*T*) under *H* = 100 Oe by the above law for *x* ≥ 0.1, and if so, to investigate the strengthening of GP with Gd doping.

**Fig. 4:**
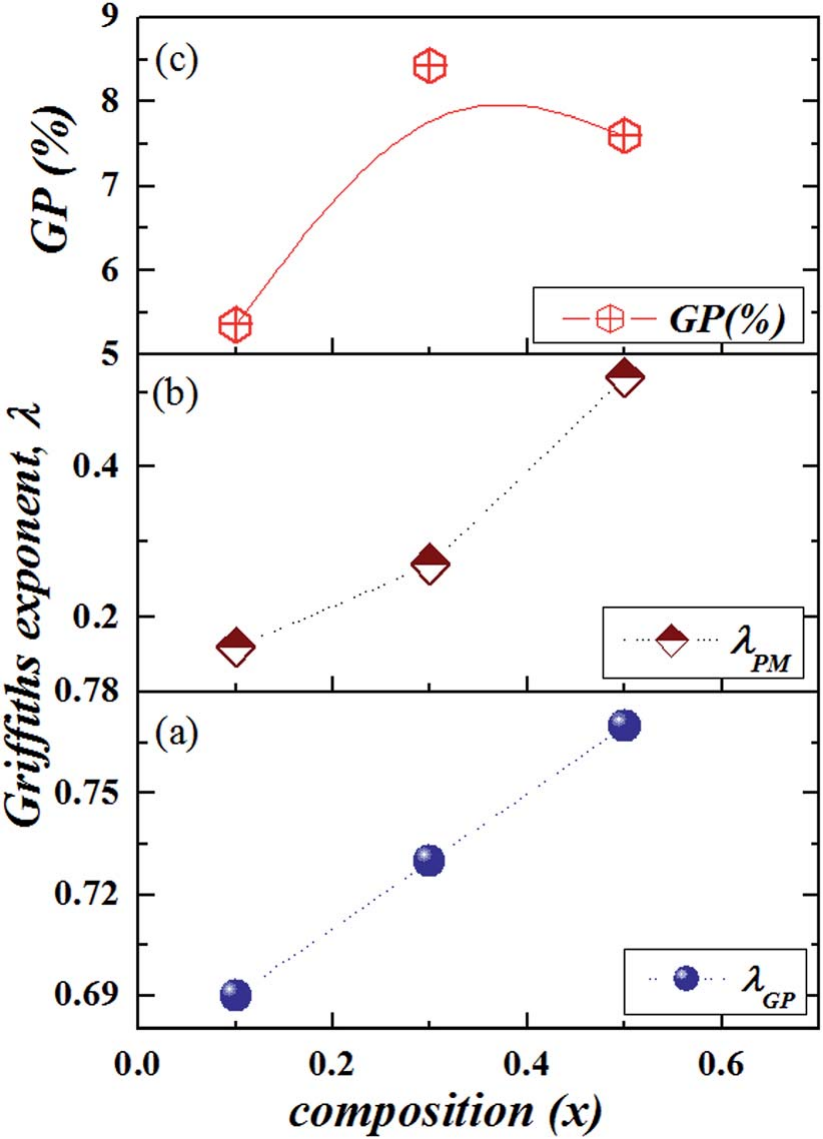
*x*-Dependence of (a) *λ*_GP_, (b) *λ*_PM_, and (c) the temperature range of Griffiths phase GP (%).

**Fig. 5:**
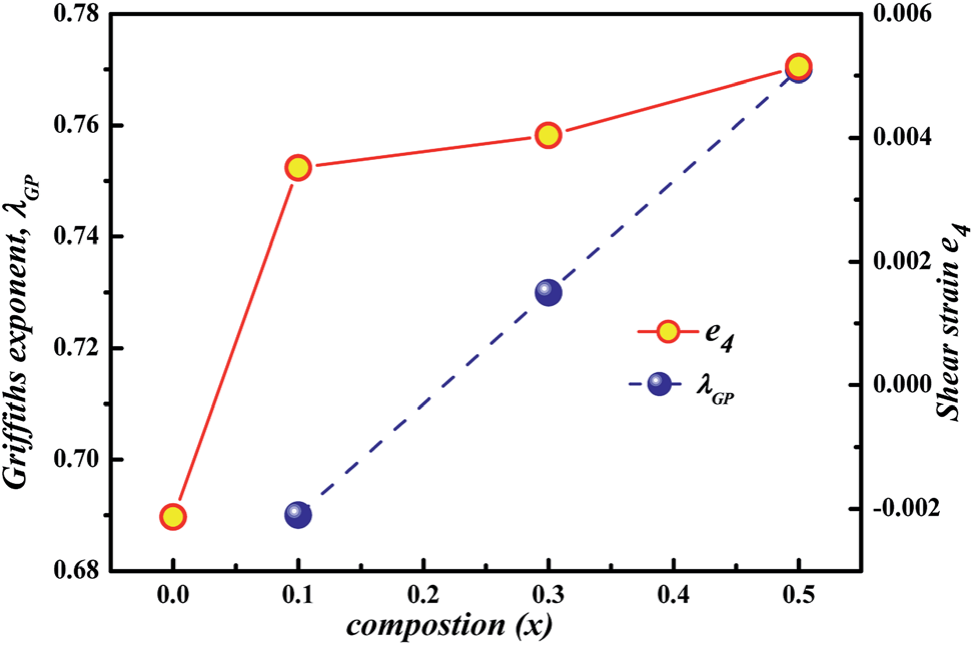
*x*-Dependence of *λ*_GP_ and shear strain *e*_4_.

To find out *λ*, *χ*^−1^*vs.* reduced temperature 
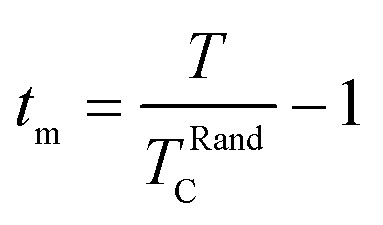
 is plotted on log_10_–log_10_ scale, and the slope of fitted straight lines [eqn (2)] both in GP regime and PM state gives *λ*_GP_ and *λ*_PM_, respectively. While specific criteria exist for determining the Griffiths temperature, *T*_G_, *i.e*., the onset of marked departures from CW behavior,^14–21^ the choice of *T*^Rand^_C_ has been less precise. It is pertinent to note that an incorrect value of *T*^Rand^_C_ in eqn (2) can lead to unphysical fitting and erroneous determination of *λ*. To estimate *T*^Rand^_C_ accurately, we have followed a method where initially *λ*_PM_ is calculated with *T*^Rand^_C_ = *T*_C_. We then adjusted the *T*^Rand^_C_ in above fitting and accepted the value for which *λ*_PM_ becomes close to zero. This is done following the fact that GP transforms into conventional PM state above *T*_G_ and the system obeys the CW law. With this *T*^Rand^_C_, we have calculated *λ*_GP_. Such fitting of eqn (2) is shown in the inset of Fig. 2(a)–(c) for *x* ≥ 0.1. The *λ*_GP_ values are plotted in Fig. 4 along with *λ*_PM_. The *x*-dependence of Griffiths temperature (*T*_G_), and the critical temperatures *T*_C1_ and *T*_C2_ are summarized in Fig. 5. Though *T*_C1_ and *T*^Rand^_C_ [inset of Fig. 2(a)–(c)] in NGSMO are close to each other for all *x*, the difference (*T*^Rand^_C_ − *T*_C1_) decreases and becomes zero with the increase of doping concentration. The exponent *λ*_GP_, obtained from the slope of the fitted straight line in the GP regime, is well consistent with the expectation from the GP model. Despite the best-fit parameters for *λ*_GP_ and *T*^Rand^_C_, the exponent *λ*_PM_ increases with *x* [Fig. 4(b)], even if it is not close to zero for *x* = 0.1. Such result shows that above *T*_G_ the PM phase is not homogeneous, this may indicate for now that the GP can extend to temperatures higher than *T*_G_.

The *λ*_GP_ deduced here is comparable to that found in a variety of doped manganites and other systems.^17–21,23^ The *λ*_GP_ in eqn (2) presents a means to measure the strength of GP. Thus, the large value of *λ*_GP_ indicates that Griffiths singularity is reasonably strong in NGSMO. Moreover, with the substitution of Gd, *λ*_GP_ increases [Fig. 4(a)] indicating a further increase in the GP properties. Surprisingly, the temperature range of GP normalized with the respective *T*_C_'s calculated as GP (%*T*_C1_) = [(*T*_G_ − *T*_C1_)/*T*_C1_] × 100 [see Fig. 4(c)] indicates a non-monotonic variation with *x* (that is to say with *σ*^2^) and presents a maximum at *x* = 0.3. On the other hand, the absence of GP for *x* = 0, yet GP is observed near half-doped.^30^ Indeed, the disorder arising from local lattice distortion is less likely to give rise to an inhomogeneous state and GP.

Despite the fact that the quenched disorder (contributed by different factors: structural or magnetic) is argued to lead to a distribution of exchange energies that causes Griffiths-like behavior in manganite systems,^14^ there appears to be a lack of consensus on the exact source of disorder responsible for the observation of GP. The experimental observation shows that the bending Mn–O–Mn bond in La_1−*x*_Ca_*x*_MnO_3_(*x* = 0.3)^14,23^ while the static J–T distortions in La_1−*x*_Sr_*x*_MnO_3_ are considered as the source of disorder inducing GP.^16^ By contrast, in La_1−*x*_Ba_*x*_MnO_3_, the quenched disorder arising from the size variance of La/Ba atoms was reported to be responsible for the development of the GP,^17^ while in (La_1−*y*_Pr_*y*_)_0.7_Ca_0.3_Mn^16/18^O_3_, a close relationship between the FM–AFM phase competition and the nucleation of the GP was observed, where the GP appears as the FM phase dominates and disappearing as the AFM phase dominates over the FM one.^31^ In fact, the Rietveld refinements carried out in our previous work,^24^ reveal that the MnO_6_ octahedra are not regular with higher dispersion in the magnetically active Mn–O–Mn bond distances and bond angles. The average 〈Mn–O〉 bond lengths show an almost negligible dependence on *x*, a result that is not surprising considering their ensitiveness to the J–T active Mn^3+^ oxidation state, which remains unchanged for this series of compounds. Such a result shows that the exact source of the quenched disorder must be only the size variance of Nd/Gd/Sr atoms.

For *x* ≥ 0.1, we come with a complicated scenario; with the increase of the quenched disorder (*σ*^2^), the *T*_G_ decreases slowly, the strength (*λ*_GP_) of the GP is enhanced, while a non-monotonic variation is obtained for the temperature range of GP. Usually, the extent of the GP-like region increases with the addition of quenched disorder.^21^ This result has some interesting consequences *vis-à-vis* the La-based manganites. The question that arises now: what is the exact mechanism behind the emergence of GP?

Based on the global phase diagram constructed by Tomioka *et al.*^32^ in the plane of the averaged ionic radius 〈*r*_A_〉 *vs.* the magnitude of quenched disorder *σ*^2^ of the Re_0.55_Sr_0.45_MnO_3_ series, we can draw the following result: since the Nd^3+^ and Gd^3+^ present a relatively similar size, the NGSMO samples should belong to the type of weakly disordered manganites. In the weakly disordered system, the accommodation strain, rather than the quenched disorder, is considered to be responsible for the intrinsic inhomogeneity or phase separation.^33^

In fact, our X-ray diffraction data shows a coexistence of two distinct orthorhombic-like phases for the higher Gd doping sample and reveals the crucial role of the shear strain *e*_4_ (the dominant strain in the region 0.1 ≤ *x* ≤ 0.5 samples) involved in the structure and its coupling with magnetic order.^24^Fig. 5 clearly shows the increase of *λ*_GP_ with shear strain, while we notice the influx of accommodation strain at the creation of GP. Furthermore, the different magnetic data show several features which are compatible with microscopic phase separation (*i.e*. phase coexistence) occurring in compounds *x* ≥ 0.1: two step magnetization, a continuous increase of *M* with high magnetic field, an irreversibility at low temperature, a metamagnetic transition and a presence of exchange bias. These features were explained according to the influence of martensitic strain on the magnetization behavior. Therefore, it implies that in the present system, the strain field might account for the electronic phase separation in a well-extended temperature range, that is, the GP above *T*_C_. This finding is in accordance with Tong *et al.*^20^ for Sm_1−*x*_Ca_*x*_MnO_3_ and in agreement with Ulyanov *et al.*^34^ for La_1−*x*_MnO_3+*δ*_ where the origin of the GP was associated with the accommodation strain. Even though it is too arbitrary to ascribe the Griffiths phase to the accommodation strain, the contribution from quenched disorders cannot be thoroughly excluded at present, since the Griffiths exponent *λ*_GP_ follows relatively the variance *σ*^2^. Consequently, more efforts from both experimental and theoretical sides are desired in order to clarify the nature and the origin of GP in these manganites.

Notwithstanding the discussion mentioned above which deals with the effect of the quenched disorder and the accommodation strain, the evolution of GP is still clearly not understood. In fact, further problems were revealed in magnetic measurements such as: (i) the coercive field [Fig. 3(d)] in the region *T*_C1_ < *T* < *T*_G_ in *x* = 0.5, verifying the existence of FM domains (ii) the pronounced temperature hysteresis for *x* = 0.5 in *χ*^−1^ [Fig. 2(d)] reveals irreversibility in the region *T*_C1_ < *T* < *T*_G_ illustrating the importance of the measurement history, (iii) the increase of *λ*_PM_ with the increase in *x* (*i.e*., for a conventional PM *λ*_PM_ = 0). Thus, we can say that it is doubtful that this behavior can be attributed to a GP.

We briefly emphasize that exact analytic description of GP appearance is reported only for specific random Ising model.^7^ While doped manganites are random three-dimensional (3D) Heisenberg, rather than Ising ferromagnetic. In addition, the criterion 0 < *λ* < 1 often considered in literature as a definite hallmark of the GP appearance^14^ was in fact predicted in only for the quantum GP in the special case of heavy fermion materials near *T* = 0.^35^ For more details, see Souza *et al.*^36^ and references therein which highlight that idealized thermodynamic model of GP is inappropriate for description of the PM state in doped manganites in general.

Souza *et al.*^36^ carried careful measurements of *χ*(*T*) in a wide *T*-interval (*T*_C_ < *T* < 705 K) revealing a strong non-linearity of the PM *χ*^−1^(*T*) in La_0.7_Ca_0.3_MnO_3_ ceramics [*e.g.* sample used by Salamon *et al.*^14^ who introduced GP paradigm in physics of doped manganites]. Souza *et al.*^36^ demonstrated that, in this case, small FM polarons (double exchange coupled pairs of Mn ions) increase in density with cooling, which changes the Curie constant. Below some characteristic T* > *T*_C_, these polarons begin to interact below T* (*T*_G_ in our case), inducing a downturn of *χ*^−1^(*T*) upon further cooling. Interestingly, authors in ref. 36 observed the same behavior in the region *T*_C_ < *T* < T* in Fig. 2(d). Indeed, Fig. 2(d) shows a distinct downturn with pronounced hysteresis in *T* illustrating the importance of the measurement history. The irreversibility with significant hysteresis in the region *T*_C1_ < *T* < *T*_G_ is suggestive of magnetic frustration. The hysteresis in *χ* reflects a different magnetic evolution of polarons depending on whether the frustrated state is approached from above T* (*i.e*., from the paramagnetic state) or below *T*_C_ (from the ferromagnetic state).^36^ Their analysis is that FM polarons play a dominant role and that the behavior in the region between *T*_C_ and T* results from frustrated FM polarons.

Recently, Rozenberg *et al.*^37^ have emphasized that the model of a determinative influence of chemical/structural disorder is more adequate for a description of the PM state than an idealized approach of thermodynamic GP. In fact, careful study of structure magnetic and resonance properties of La_0.9_Sr_0.1_MnO_3_ crystal definitely evidences the presence of macroscopic (two structural phases) and mesoscopic (spatially inhomogeneous Sr-dopant distribution) structural disorder in this object,^37^ as observed for our sample *x* = 0.5 in NGSMO.^24^ Such disorder changes the nature of the FM–PM transition and induces FM and PM phase coexistences far above the *T*_C_. The system of mesoscopic FM correlated clusters having a wide distribution of their local *T*_C_'s appears due to the existence of the nanometer sized La- and Sr-enriched regions.^38^ An interaction between such FM correlations causes a downturn of *χ*^−1^(*T*) on cooling (Fig. 1 and ref. 36) and explains such feature without using the Griffiths scenario.

The phenomenon of irreversibility already observed by Souza *et al.*^36^ reflects a different magnetic evolution of polarons according to whether the frustrated state is approached above *T*_G_ (the PM state) or below *T*_C_ (the FM state). Their analysis is that the FM polarons play a dominant role and the behavior in the region between *T*_C_ and *T*_G_ results from frustrated FM polarons. As well, the existence of two structural phases [ref. 24] can be explained by Rozenberg *et al.*^37^ by the presence of a chemical/structural disorder and not GP although we have a correct fit of the value of *λ*, (0 < *λ* < 1). Therefore, we cannot confirm that we don't have a GP.

To sum up, the PM phase is governed by large magnetic clusters in the *T*_C1_ < *T* < *T*_G_ region associated with the AFM coupled with GP by frustrated FM polarons resulting from the structural/chemical disorder.

Now we return to the relevant results of the heavily Gd-doped sample (*x* = 0.5). Hysteresis and the coercive field in the region *T*_C1_ < *T* < *T*_G_, which may be originated from a peculiar magnetic domain structure. Particularly, these domains correspond to a magnetic spin polarons following ref. 36. In fact, the hysteresis signifies polaron–lattice coupling. Generally, the polaronic state in manganese perovskites is a consequence of a strong electron–phonon coupling of the Jahn–Teller (JT) active Mn^3+^ ions. On the other hand, the magnetic results of *χ*^−1^(*T*) show pieces of evidence that is in agreement with the GP (*i.e*. preformation of FM clusters above the FM long-range ordering temperature *T*_C1_).

Hence, our interpretation can be based on electronic phase separation developed at the nanometer scale, where FM clusters nanodomains due to GP are intrinsically AFM exchange coupled with correlated JT polarons. This model seems to be much more realistic for description of the PM state in *x* = 0.5 sample than an idealized Griffiths phase approach. Theoretically, it has been shown that FM and nm size AFM phases coexist even in the absence of A-site disorder but at a sufficiently large electron–phonon coupling.^39^ Correlated polarons, associated with orbital polarization of Mn^3+^ states and corresponding static Jahn–Teller (JT) distortions of MnO_6_ octahedrons, may contribute to the nm-scale phase separation.^40^ Correlated polarons have been experimentally detected by neutron scattering and X-ray diffraction in the form of short-range ordered lattice superstructures of CE-type with a correlation length of about 1–2 nm.^41,42^

Finally, we think that, by means of isovalent substitution of a large Nd^3+^ cation by a smaller Gd^3+^ in the hole-doped Nd_0.55_Sr_0.45_MnO_3_ (NSMO), an increase of electron–phonon interaction favors the tendency of phase competition. Thus, The FM clusters ground state for *x* = 0 progressively transforms with increasing ‘*x*’ into a mixture of FM and AFM phases coupled with correlated Jahn–Teller polarons.

**Fig. 6:**
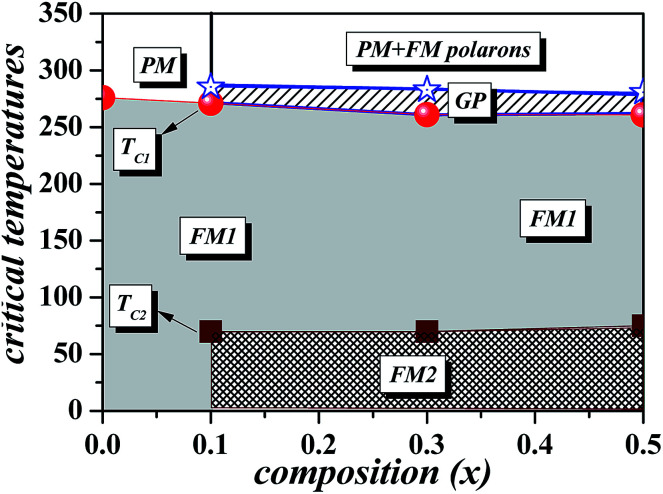
Magnetic phase diagram of (Nd_1−*x*_Gd_*x*_)_0.55_Sr_0.45_MnO_3_ (*x* = 0, 0.1, 0.3 and 0.5). PM, GP, and FM denote the paramagnetic, Griffiths, and ferromagnetic phase, respectively.

Using the experimentally determined *T*_C1_(*x*),^24^*T*_C2_(*x*),^24^ and *T*_G_(*x*) dependence, we reconstruct the (*x*, *T*) magnetic phase diagrams of the (Nd_1−*x*_Gd_*x*_)_0.55_Sr_0.45_MnO_3_ system [Fig. 6]. From Fig. 6 we notice that, the evolution of the GP with the composition (*x*) is very analogous to those reported in the doped manganite La_1−*x*_Sr_*x*_MnO_3_ (ref. 16) and La_1−*x*_Ba_*x*_MnO_3_ (ref. 39) and can be comparable with the *T*–*p* phase diagram from the Griffiths model.^16,39^ Once more, these features strongly indicate that the clustered state in the NGSMO can be well described by the GP. Hence, based on the above discussion, the PM state in this case can be governed by the largest magnetic cluster in the region *T*_C1_ < *T* < *T*_G_ owing to the GP antiferromagnetically coupled by correlated small frustrated ferromagnetic polarons resulting from chemical/structural disorder whose concentration increases upon cooling towards the Curie temperature. When the temperature declines [ref. 24], there is a FM1 state: it is a ferromagnetic state with the presence of two Curie temperatures for *x* > 0. This ferromagnetic state keeps itself even when the temperature is lowered but just for *x* = 0. for *x* > 0, there is an appearance of a state FM2 which becomes more and more inhomogeneous with the composition *x*. This state becomes an AFM/ferrimagnetic and FM/ferrimagnetic region.^24^

## IV. Conclusion

The magnetic properties of perovskite rare-earth manganites (Nd_1−*x*_Gd_*x*_)_0.55_Sr_0.45_MnO_3_ with *x* = 0, 0.1, 0.3 and 0.5 have been investigated. A typical character of the Griffiths phase was found in the inverse magnetic susceptibility for *x* ≥ 0.1 above Curie temperature (*T*_C_). The estimated susceptibility exponent (*λ*_GP_), the GP temperature (*T*_G_), and the temperature range of GP (GP (%)) show that the strain field, in addition to quenched disorders quantified by the variance *σ*^2^, is prerequisite to the formation of GP. This result links the Griffiths phase to the accommodation strain. However, the increase of *λ*_PM_ with the increase in *x*, and the pronounced hysteresis for *x* = 0.5 in the region *T*_C1_ < *T* < *T*_G_ are at odds with the Griffiths model. With this scenario, the downturn anomalies on *χ*^−1^(*T*) appear most likely due to the presence of magnetic polarons, whose concentration increases upon cooling towards *T*_C_. Yet, the *χ*^−1^(*T*) is well fitted with the power law in eqn (2) according to the model of the GP. The magnetic results for the heavily Gd-doped sample (*x* = 0.5) are discussed within a phase separation scenario with coexisting FM nanodomains antiferromagnetically coupled by correlated polarons.

## Conflicts of interest

There are no conflicts to declare.

## Supplementary Material
